# The Impairment of Endothelial Autophagy Accelerates Renal Senescence by Ferroptosis and NLRP3 Inflammasome Signaling Pathways with the Disruption of Endothelial Barrier

**DOI:** 10.3390/antiox13080886

**Published:** 2024-07-23

**Authors:** Jin Won Kim, Sun Ah Nam, Eun-Sil Koh, Hyung Wook Kim, Sua Kim, Jin Ju Woo, Yong Kyun Kim

**Affiliations:** 1 Department of Cell Death Disease Research Center, College of Medicine, The Catholic University of Korea, Seoul 06591, Republic of Korea; 2 Department of Internal Medicine, College of Medicine, The Catholic University of Korea, Seoul 06591, Republic of Korea; 3 Department of Internal Medicine, College of Medicine, The Catholic University of Korea, St. Vincent’s Hospital, Suwon 16247, Republic of Korea

**Keywords:** autophagy, aging, kidney, liproxstatin-1

## Abstract

Autophagy is a cellular process that degrades damaged cytoplasmic components and regulates cell death. The homeostasis of endothelial cells (ECs) is crucial for the preservation of glomerular structure and function in aging. Here, we investigated the precise mechanisms of endothelial autophagy in renal aging. The genetic deletion of Atg7 in the ECs of Atg7*^flox/flox^*;Tie2-Cre mice accelerated aging-related glomerulopathy and tubulointerstitial fibrosis. The EC-specific Atg7 deletion in aging mice induced the detachment of EC with the disruption of glomerular basement membrane (GBM) assembly and increased podocyte loss resulting in microalbuminuria. A Transwell co-culture system of ECs and kidney organoids showed that the iron and oxidative stress induce the disruption of the endothelial barrier and increase vascular permeability, which was accelerated by the inhibition of autophagy. This resulted in the leakage of iron through the endothelial barrier into kidney organoids and increased oxidative stress, which led to ferroptotic cell death. The ferritin accumulation was increased in the kidneys of the EC-specific Atg7-deficient aging mice and upregulated the NLRP3 inflammasome signaling pathway. The pharmacologic inhibition of ferroptosis with liproxstatin-1 recovered the disrupted endothelial barrier and reversed the decreased expression of GPX4, as well as NLRP3 and IL-1β, in endothelial autophagy-deficient aged mice, which attenuated aging-related renal injury including the apoptosis of renal cells, abnormal structures of GBM, and tubulointerstitial fibrosis. Our data showed that endothelial autophagy is essential for the maintenance of the endothelial barrier during renal aging and the impairment of endothelial autophagy accelerates renal senescence by ferroptosis and NLRP3 inflammasome signaling pathways. These processes may be attractive therapeutic targets to reduce cellular injury from renal aging.

## 1. Introduction

A rise in human life expectancy in recent decades has led to an increase in the prevalence of elderly individuals [1]. Aging leads to a progressive deterioration in functioning and structural changes in most organs [1]. The kidney is particularly susceptible to age-related damage [2]. Aging is a major risk factor for acute kidney injury and chronic kidney disease, which not only decreases the quality of life but also survival in the elderly population [2,3,4,5]. An understanding of cellular and molecular mechanisms in kidney aging could provide therapeutic strategies.

Aging induces the accumulation of damaged proteins, lipids, organelles, and mitochondria, which are physiological causes of the age-associated impairment of cellular function and limit the capacity for regeneration [2]. Efficient cellular mechanisms to degrade accumulated protein aggregates and altered organelles are required to maintain cellular homeostasis during aging. Autophagy is an evolutionarily conserved lysosomal-mediated cellular process that functions in the degradation of damaged organelles, protein aggregates, and other macromolecules in the cytoplasm and regulates cell death under normal physiological conditions as well as pathological conditions [6,7]. Autophagy has been reported to play an important role in the maintenance of cellular homeostasis during aging [8,9].

In natural kidney aging, the steady-state (basal) autophagy seems to be different by the cell types constituting the kidney [10]. A previous study reported that basal autophagy in proximal tubular cells was almost absent in young mice and was higher in aged mice. Basal autophagy in podocytes was constitutively high in young mice and unchanged in aged mice [9]. Different forms of basal autophagy and autophagic activity in response to metabolic stress such as calorie restriction were decreased in aging mice compared to young mice [9]. Previous studies showed the altered mitochondrial morphology and accumulation of age-associated proteins, such as SQSTM1, in accordance with a decrease in autophagy activity, in aging experimental models [11]. In aging kidney tubular cells, the accumulation of autophagosome was also observed, which seems to be due to lysosomal dysfunction or abnormal autophagosome maturation rather than decreased autophagosome biogenesis.

Among kidney cells, the endothelial cells (ECs) regulate blood flow and vascular permeability. The homeostasis of glomerular ECs is crucial for the preservation of glomerular structure and function, preventing vascular structural and functional changes from renal disease such as diabetic nephropathy [12,13,14]. Glomerular ECs exhibit substantial autophagic activity, which plays an essential role in maintaining their homeostasis [15,16].

In the aging kidney, EC senescence induces vascular structural and functional changes that enhance thrombosis, inflammation, and atherosclerosis through the impairment of vessel tone, angiogenesis, and vascular integrity. These changes can contribute to the development and progression of cardiovascular diseases. Basal autophagy in glomerular ECs is low, but the autophagic activity in response to calorie restriction is high in young mice, which may keep the integrity of glomerular capillaries by protecting glomeruli from oxidative stress [16]. The precise mechanism of endothelial autophagy in kidney aging remains unclear.

Ferroptosis is an iron-dependent regulated cell death characterized by the production of reactive oxygen species (ROS) from accumulated iron and lipid peroxidation [17,18]. Ferroptosis is mainly caused by deficits in the production of reduced glutathione or by the dysfunction of glutathione peroxidase 4 (GPX4), which are ROS eliminators [19].

The NOD-like receptor pyrin domain-containing 3 (NLRP3) inflammasome signaling pathway regulates a variety of host innate immune defense pathways in response to pathogens or damage-associated molecular patterns by microbial and nonmicrobial stimuli [20]. NLRP3 is a member of the NOD-like receptor (NLR) family involved in the innate immune response [21]. NLRP3 forms a protein complex, the inflammasome, which induces caspase-1 activation that results in the maturation and secretion of proinflammatory cytokines such as interleukin (IL)-1β and IL-18 [20].

We hypothesized that endothelial autophagy interacts with the ferroptosis and NLRP3 inflammasome signaling pathway and regulates renal senescence. Using a Transwell co-culture system of EC and kidney organoids as an in vitro model and EC-specific Atg7-deficient mice as an in vivo model, we found that a deficiency in endothelial autophagy induced the disruption of the endothelial barrier and increased vascular permeability, the leakage of iron, and oxidative stress in kidney cells. This accelerated ferroptosis and activated the NLRP3 inflammasome signaling pathway during renal senescence.

## 2. Materials and Methods

### 2.1. Animals and Treatment

Atg7*^flox/flox^* mice were crossed with Tie2-Cre mice (Jackson ImmunoResearch Laboratories, West Grove, PA, USA) to generate endothelial cell-specific Atg7 knockout mice (Atg7*^flox/flox^*;Tie2-Cre mice). Atg7*^flox/flox^* littermates served as controls. All mice were crossed on a C57BL6 background, and only male mice were used in the study.

Five groups of animals were included in this study: young WT mice (3 months; *n* = 4); young Atg7*^flox/flox^*;Tie2-Cre mice (3 months; *n* = 4); old WT mice (18 months; *n* = 4); old Atg7*^flox/flox^*;Tie2-Cre mice (18 months; *n* = 4); and old Atg7*^flox/flox^*;Tie2-Cre mice treated with liproxstatin-1 (SML1414, Sigma-Aldrich, St. Louis, MO, USA) (18 months; *n* = 4). In the fifth group, liproxstatin-1 (10 mg/kg) was injected intraperitoneally into old Atg7*^flox/flox^*;Tie2-Cre mice once every two days for a month.

Mice were sacrificed, and their kidneys were briefly perfused with phosphate-buffered saline (PBS, pH 7.4) to remove any residual blood. This was followed by a 10 min perfusion with a periodate–lysine–2% paraformaldehyde solution. After perfusion, the kidneys were excised and sliced into 1–2 mm thick sections, which were then immersed in the same fixative overnight at 4 °C for further fixation. Following this, the kidney slices were rinsed in PBS, dehydrated through a graded ethanol series, and embedded in paraffin.

### 2.2. Immunohistochemical Immunofluorescence and Analysis

Some kidney sections were processed and stained with periodic acid-Schiff (PAS) or Masson’s trichrome stain. Other sections were processed for post-embedding immunohistochemistry analysis. After deparaffinization, the sections were hydrated and incubated with 0.5% Triton X-100/PBS solution for 30 min and then they were washed with PBS three times. The nonspecific binding sites were blocked with normal donkey serum diluted 1:10 in PBS for 1 h, and then the sections were incubated overnight at 4 °C with a primary antibody. After being rinsed in PBS, the sections were incubated in peroxidase-conjugated anti-mouse or anti-rabbit IgG (Jackson ImmunoResearch Laboratories) for 1 h. The sections were incubated with a mixture of 0.05% 3,3′-diaminobenzidine containing 0.01% H_2_O_2_ at room temperature until a brown color was visible. They were then washed with Tris buffer (pH 7.6), counterstained with hematoxylin, and observed under light microscopy. The sections were scanned and automatically digitized using a Leica SCN400 (Leica, Wetzlar, Germany). They were then analyzed using Tissuemorph/DP software version 5.3.1.1723 (Visiopharm, Hørsholm, Denmark).

For immunofluorescence analysis, the kidney sections were hydrated using a series of graded ethanol solutions and rinsed with tap water. The sections were then treated with a retrieval solution (pH 6.0), followed by 0.5% Triton X-100 in PBS, and blocked with normal donkey serum. Next, they were incubated with primary antibodies overnight at 4 °C. The following day, after washing with PBS, the tissue sections were incubated with the appropriate fluorescence-labeled secondary antibodies and mounted in a Vectashield mounting medium (Vector Laboratories, Burlingame, CA, USA). Images were captured using a Zeiss LSM 700 confocal microscope (Carl Zeiss, Jena, Germany).

### 2.3. Western Blot Analysis

The kidneys were homogenized in boiling lysis buffer (1% SDS, 1 mM sodium orthovanadate, and 10 mM Tris, pH 7.4) and the protein concentration was determined with a BCA protein assay kit (Pierce Biotechnology Inc., Rockford, IL, USA). Equal amounts of the protein were separated using SDS–polyacrylamide gel electrophoresis. The gel was then transferred onto an NC membrane. For immunodetection, the blots were incubated overnight in PBS containing 0.1% Tween-20, 5% skim milk, and the primary antibody. The blots were washed and then incubated with a secondary antibody conjugated to horseradish peroxidase (Jackson ImmunoResearch Laboratories), and the blots were visualized using a Western blotting luminol reagent kit (Santa Cruz Biotechnology, Santa Cruz, CA, USA).

### 2.4. Antibodies

The antibodies used in this study were as follows: Atg7 (A2856, Sigma-Aldrich), TGF-β (MA13240, R&D Systems, Minneapolis, MN, USA), α-SMA (A2547, Sigma-Aldrich), NLRP3 (A27461511, Adipogen, San Diego, CA, USA), caspase-1 (sc-56036, Santa Cruz Biotechnology, Santa Cruz, CA, USA), IL-β (12242, Cell Signaling Technology, Beverly, MA, USA), 8-OHdG (MOG-0209, JaICA, Shizuoka, Japan), ferritin light chain (ab69090, Abcam, Cambridge, UK), ferritin heavy chain (ab65080, Abcam), CD31 (AF3628, R&D Systems), WT1 (ab89901, Abcam), C-Myc (ab32072, Abcam), 4-hydroxynonenal (HNE, MHN-0209, JaICA), β-actin (A5441, Sigma-Aldrich), GPX4 (ab125066, Abcam), GAPDH (sc-32233, Santa Cruz Biotechnology), and VECAD (555661, BD Biosciences, San Jose, CA, USA). Apoptosis was detected using an ApopTag Peroxidase In Situ Apoptosis Detection Kit (Millipore, Billerica, MA, USA).

### 2.5. Electron Microscopic Analysis

To observe autophagy and the ultrastructural changes in mitochondria, we conducted a conventional transmission electron microscopic (EM) study. Kidney block samples were fixed in a solution of 2% paraformaldehyde and 2.5% glutaraldehyde in 0.1 M phosphate buffer overnight at 4 °C. After washing with 0.1 M phosphate buffer, the samples were postfixed with 1% osmium tetroxide in the same buffer for 1 h at 4 °C. Subsequently, the samples were dehydrated through a series of graded ethanol solutions, transitioned through acetone, and embedded in Epon 812.

Ultrathin sections (70~80 nm) were obtained with an ultramicrotome (Ultracut UCT, Leica, Austria). Ultrathin sections were double stained with uranyl acetate and lead citrate, and they were examined with a transmission electron microscope (JEM 1010, JEOL, Tokyo, Japan) at 60 kV. For quantitative determination, 20 fields at low magnification (×6000) were randomly selected from each section of the cortex, and the number of autophagosomes per 100 μm^2^ was evaluated.

### 2.6. Urine Analysis

The mice were placed in individual mouse metabolic cages to collect urine for 24 h. Urine albumin concentrations were measured using a mouse albumin ELISA kit (Alpco Diagnostics, Salem, NH, USA) in mice from each group according to the manufacturer’s protocols.

### 2.7. Kidney Organoid Differentiation and Transwell Co-Culture System of ECs and Kidney Organoids

We used WTC11 cells obtained from The Catholic University of Korea. Cells were used between passages 30 and 60. Kidney organoid differentiation was performed as described previously [22,23]. In summary, human pluripotent stem cells (hPSCs) were seeded at a density of 5000 cells per well in a 24-well plate using mTeSR1 medium (Stem Cell Technologies, Vancouver, BC, Canada) supplemented with 10 µM Y27632 (LC Laboratories, Woburn, MA, USA) on glass plates (LabTek, Rochester, NY, USA) coated with 0.1% GelTrex (Thermo Fisher Scientific, Waltham, MA, USA) on day 1. On day 2, the medium was replaced with 1.5% GelTrex in mTeSR1, followed by mTeSR1 on day 3. On day 4, the medium was switched to RPMI (Thermo Fisher Scientific) with 11 µM CHIR99021 (Tocris Bioscience, Bristol, UK), and on day 5, RPMI with B27 supplement (Thermo Fisher Scientific) was used. The cells were fed every 2–3 days to promote kidney organoid differentiation. On Day 18, kidney organoids and human umbilical vein endothelial cells (HUVECs) were cultured separately using a 12-well Transwell system (0.4 μm pore; Corning Inc., Corning, NY, USA). Kidney organoids were plated onto the bottom of a 12-well plate and HUVECs were seeded onto the insert at a density of 1 × 10^5^ cells/mL for 24 h. After adding 5 mM H_2_O_2_ and 500 μM Fe^2+^ to the Transwell and incubating for 24 h, 2 μM liproxstatin-1 and 3 mM 3MA was added and left for 24 h. The selected dose was determined according to cell viability (Figure S1A,B).

### 2.8. Measurement of Transepithelial Electrical Resistance

We monitored the integrity of the paracellular pathway of HUVEC monolayers to small ions by the measurement of transepithelial electrical resistance (TER) using an EVOMTM (World Precision Instruments, Sarasota, FL, USA). HUVECs were seeded onto Transwell filters (diameter 12 mm, pore size 0.4 μM, Corning) to measure TER after exposure to Fe^2+^ (500 μM), H_2_O_2_ (5 mM), liproxstatin-1 (2 μM), and 3MA (3 mM) in an apical chamber for 24 h. TER was normalized to the area of the filter after the removal of background resistance using a blank filter on which cells were not seeded and which contained only culture medium. TER was thus measured as Ω·cm^2^ and was zero when determined without cells. TER was calculated as ohms × cm^2^ (Ω·cm^2^) after subtracting values for the resistance of the membrane support alone.

### 2.9. Transwell Permeability Assay

HUVECs were seeded 1 × 10^5^ cells/well into Transwell inserts (diameter 12 mm, pore size 0.4 μM, Corning) and exposed to Fe^2+^ (500 μM), H_2_O_2_ (5 mM), liproxstatin-1 (2 μM), and 3MA (3 mM) in the upper compartment for 24 h. On the following day, the supernatant was removed and fluorescein isothiocyanate (FITC) dextran (2 mg/mL; 70 kDa, Sigma-Aldrich) was added to the Transwells. After 2 h of incubation, the fluorescence of the FITC-dextran in the lower compartment was measured in a microplate reader at 490/520 nm.

### 2.10. Iron Assay

To measure cellular iron levels, we used a colorimetric Iron Assay Kit (ab83366, Abcam). HUVECs were seeded 5 × 10^4^ cells/well into Transwell inserts (diameter 24 mm, pore size 0.4 μM, Corning), and kidney organoids reseeded into the lower compartment. HUVECs were exposed to Fe^2+^ (500 μM), H_2_O_2_ (5 mM), liproxstatin-1 (2 μM), and 3MA (3 mM) in the upper compartment for 24 h. In the lower compartment, 200 μL of iron assay buffer was added to kidney organoids on ice for homogenization and centrifuged at 4 °C and 12,000 rpm for 10 min. The supernatant was used following the manufacturer’s instructions. A microplate reader was used to measure the absorbance of each well at 593 nm.

### 2.11. Measurement of Reactive Oxygen Species

The levels of ROS were measured with 2′,7′-dichlorofluorescein diacetate (DCFDA) Cellular ROS Assay Kit (ab113851, Abcam). HUVECs were seeded into Transwell inserts and kidney organoids were reseeded into the lower compartment. HUVECs in the upper compartment were exposed to Fe^2+^ (500 μM), H_2_O_2_ (5 mM), liproxstatin-1 (2 μM), and 3MA (3 mM). After 24 h, the medium was removed, the kidney organoids were washed with PBS, and ROS were measured by using a microplate reader according to the manufacturer’s instructions.

### 2.12. Three-Dimensional Cell Viability Assay and Live/Dead Cell Staining

HUVECs were seeded 5 × 10^4^ cells/well into Transwell inserts (diameter 24 mm, pore size 0.4 μM, Corning), and kidney organoids were reseeded into the lower compartment. HUVECs were exposed to Fe^2+^ (500 μM), H_2_O_2_ (5 mM), liproxstatin-1 (2 μM), and 3MA (3 mM) in an upper compartment for 24 h. For the 3D cell viability assay, an equal volume of Cell Titer-Glo 3D Reagent (Promega, Madison, WI, USA) was added to the kidney organoid culture medium in each well of a 96-well plate. The contents were mixed for 5 min to induce cell lysis, followed by incubation at room temperature for 25 min before recording the luminescence. For live/dead cell staining, the live/dead viability/cytotoxicity kit reagents (ethidium homodimer 2 mM, calcein-AM 4 mM; Thermo Fisher Scientific) were added to the kidney organoids and HUVECs for 1 h in the incubator after a single PBS wash. The cells were then washed twice with PBS, and z-stack fluorescence images were captured using a fluorescence microscope.

### 2.13. Real-Time Quantitative PCR Analysis

Kidney organoid samples were collected, and total RNA was extracted using the RNAiso Plus Kit (Takara, Tokyo, Japan). Complementary DNA was synthesized with the Maxima First Strand cDNA Synthesis Kit for RT-qPCR (Thermo Fisher Scientific). Gene expression was assessed with the Power SYBR Green PCR Master Mix (Applied Biosystems, Foster City, CA, USA) using real-time PCR (Applied Biosystems). The qRT-PCR was performed in triplicate, and the relative mRNA expression levels were calculated using the 2^−ΔΔCt^ method. The specific primers used were as follows: Aifm2, F-5′ GCAAAGCGTTTGAGAGCAGA and R-5′ ACAGTCACCAATGGCGTAGA; SLC7A11, F-5′ GGTCAGAAAGCCTGTTGTGT and R-5′ TCATGGAGCCAAAGCAGGAG; GAPDH, F-5′ AGGGCTGCTTTTAACTCTGGT and R-5′ CCCCACTTGATTTTGGAGGGA.

### 2.14. Statistics

Values are presented as the means ± SEM. Data were compared between groups using a Mann–Whitney test or Kruskal–Wallis test as appropriate. *p*-values less than 0.05 were considered significant. All statistical analyses were performed using SPSS 16.0 software (Chicago, IL, USA).

## 3. Results

### 3.1. EC-Specific Atg7 Deletion Accelerated Aging-Related Structural Changes and Fibrosis in Kidneys

**Figure 1:**
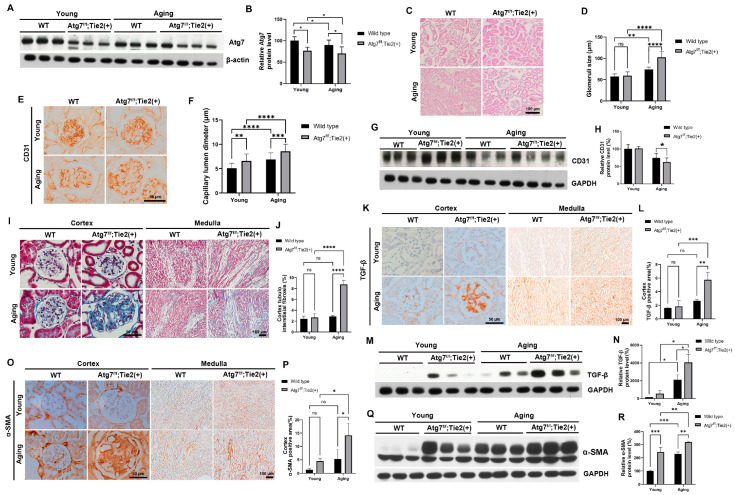
Increase in renal fibrosis in aging kidneys of Atg7*^flox/flox^*;Tie2-Cre*^+^* mice. (**A**) Representative immunoblots and densitometry for the expression of Atg7. (**B**) Relative protein level of Atg7 (%). (**C**) Representative images of H&E staining from wild-type and Atg7*^flox/flox^*;Tie2-Cre*^+^* mice. Scale bars, 100 μm. (**D**) Quantification of glomeruli diameters (µm). (**E**) Representative 3,3′-diaminobenzidine (DAB) staining for CD31. Scale bars, 50 μm. (**F**) Quantification of capillary lumen diameters (µm). (**G**) Representative immunoblots and densitometry for the expression of CD31. (**H**) Relative protein levels of CD31 (%). (**I**) Masson’s trichrome staining from wild-type and Atg7*^flox/flox^*;Tie2-Cre mice, showing the cortex and medulla with increased extracellular matrix deposition in the aging Atg7*^flox/flox^*;Tie2-Cre*^+^* mouse. (**J**) Quantification of tubulointerstitial fibrosis (%). (**K**) Representative DAB staining for TGF-β. (**L**) Quantification of TGF-β positive areas (%). (**M**) Representative immunoblots and densitometry for expression of TGF-β. (**N**) Relative protein level of TGF-β (%). (**O**) Representative DAB staining for α-SMA. (**P**) Quantification of α-SMA-positive areas (%). (**Q**) Representative immunoblots and densitometry for expression of α-SMA. (**R**) Relative protein levels of α-SMA (%). Scale bars, 50 μm for cortex and 100 μm for medulla. Values are means ± SEM. *, *p* < 0.05; **, *p* < 0.01; ***, *p* < 0.001; ****, *p* < 0.0001, ns: not significant.

To investigate the structural and functional role of endothelial autophagy in the aging kidney, we generated conditional knockout mice (Atg7*^flox/flox^*;Tie2-Cre^+^) in which Atg7 was genetically ablated in ECs specifically. The protein expression of Atg7 from Western blot analyses of whole kidney lysates was significantly decreased in the kidneys of Atg7*^flox/flox^*;Tie2-Cre^+^ mice compared with those of WT mice (Figure 1A,B). This finding confirmed an efficient deletion of Atg7 in the kidney of Atg7*^flox/flox^*;Tie2-Cre^+^ mice. No obvious histologic phenotype was observed between the young kidneys of Atg7*^flox/flox^*;Tie2-Cre^+^ mice and the young kidneys of WT mice (Figure 1C). The protein expression of Atg7 is decreased in aging mice compared to young mice (Figure 1A). This finding implies that Atg7 is a natural aging process, which is consistent with a previous study [24].

We next examined the effects of the EC-specific deletion of Atg7 on the structural changes in aging kidneys. Glomerular hypertrophy and the dilation of capillary lumens are typical structural changes in aging kidneys [25,26]. The size of the glomerulus and diameter of the capillary lumens in the glomerulus were increased in the aging kidneys of WT mice compared with those of young WT mice and substantially increased in the aging kidneys of Atg7*^flox/flox^*;Tie2-Cre^+^ mice (Figure 1C–F). The protein expression of CD31 was decreased in the aging kidneys of Atg7*^flox/flox^*;Tie2-Cre^+^ mice (Figure 1G,H). These findings indicate that autophagy deficiency in ECs accelerated aging-induced structural changes in the kidneys.

Renal fibrosis is a typical histologic feature of aging [27,28]. Therefore, we also examined the effects of an EC-specific deletion of Atg7 on fibrosis in aging kidneys. Masson’s trichrome staining revealed increased extracellular matrix (ECM) deposition within the glomerulus and tubulointerstitium in aging WT mice compared with those of young mice, and this deposition was substantially increased in the aging kidneys of Atg7*^flox/flox^*; Tie2-Cre^+^ mice (Figure 1I,J). Transforming growth factor-β (TGF-β) is a major cytokine mediating renal fibrosis by inducing the production of ECM proteins [21,29]. The upregulation of TGF-β contributes to impaired renal function in chronic kidney disease [20,30,31]. Alpha-smooth muscle actin (α-SMA) is a marker protein of smooth muscle cells and myofibroblasts. The mesangial cell activation characterized by the induction of α-SMA expression promotes the deposition of the ECM and glomerulosclerosis [32,33]. Immunohistochemical staining and Western blot analyses showed that the expression levels of TGF-β and α-SMA substantially increased in the mesangium within the glomerulus and tubulointerstitium in the medulla in the aging kidneys of Atg7*^flox/flox^*;Tie2-Cre^+^ mice compared with those of aging WT mice (Figure 1K–R). These findings indicate that autophagy deficiency in ECs accelerated renal fibrosis by the induction of TGF-β in a process that corresponds to aging.

### 3.2. EC-Specific Atg7 Deletion in Aging Mice Induced the Detachment of EC with the Disruption of GBM Assembly and Increased Podocyte Loss Resulting in Microalbuminuria

**Figure 2:**
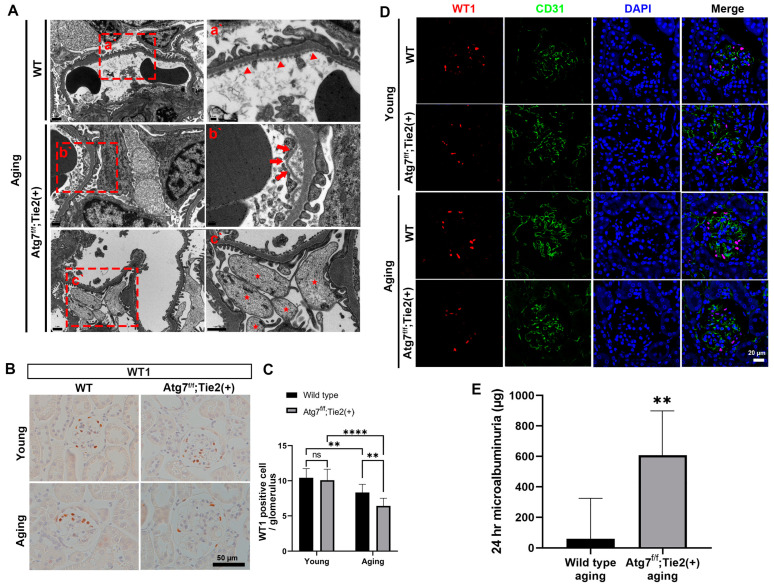
Loss of podocytes, the deterioration of slit diaphragms, and the accumulation of ferritin by EC-specific autophagy deletion in the renal aging of Atg7*^flox/flox^*;Tie2-Cre^+^ mice. (**A**) Representative TEM images showing the structural integrity of slit diaphragms in an aging WT mouse kidney (**a’**, red arrowhead) and the lamina rara interna detached from the lamina densa in some portions of the GBM (**b’**, red arrow) and apoptotic podocytes (**c’**, red asterisk) in the aging kidneys of Atg7*^flox/flox^*;Tie2-Cre^+^ mice. (**B**) Representative 3,3′-diaminobenzidine (DAB) staining for WT1. Scale bars, 50 μm. (**C**) Quantification of WT1-positive areas (%). (**D**) Representative immunofluorescent staining for WT1 (red) and CD31 (green). Scale bars, 20 μm. (**E**) Measurement of microalbuminuria in urine for 24 h from aging wild-type and aging Atg7f/f;Tie2-Cre+ mice. Values are means ± SEM. **, *p* < 0.01; ****, *p* < 0.0001, ns: not significant.

We next investigated the effect of EC-specific autophagy deletion on the ultrastructural alteration of slit diaphragms in the aging kidney by EM analysis. In the aging kidneys of Atg7*^flox/flox^*;Tie2-Cre^+^ mice, EC and the lamina rara interna in some portions of the GBM were detached from the lamina densa (b’ in Figure 2A), whereas the slit diaphragm was intact in the aging kidneys of WT mice (a’ in Figure 2A).

Endothelial–podocyte crosstalk is known to be essential for renal development and glomerular homeostasis [34]. In early diabetic kidney disease, glomerular endothelial cell dysfunction resulting from the loss of the endothelial surface layer may lead to podocyte damage and loss, which further exacerbates glomerular injury by the disruption of vascular endothelial growth factor (VEGF) signaling in podocytes, thus forming a vicious cycle. We examined whether autophagy deficiency in ECs influenced podocyte homeostasis in renal aging. Interestingly, EM analysis revealed that the widespread distribution of apoptotic podocytes was observed in the aging kidneys of Atg7*^flox/flox^*;Tie2-Cre^+^ mice (c’ in Figure 2A). WT1 immunohistochemical staining (Figure 2B,C) and immunofluorescence confocal microscopy (Figure 2D) showed that podocyte loss was exacerbated in the aging kidneys of Atg7*^flox/flox^*;Tie2-Cre^+^ mice compared with those of aging WT mice.

Microalbuminuria is considered an endothelial (microvascular) injury and a relevant marker of kidney disease and a risk for its progression [35]. A prospective large human cohort study showed that microalbuminuria is increased in aging people [36]. Given that the structure of slit diaphragms in the aging kidneys of Atg7*^flox/flox^*;Tie2-Cre^+^ mice had deteriorated, we examined the microalbuminuria. Levels of microalbumin from urine collected at 24 h were found to increase in the aging Atg7*^flox/flox^*;Tie2-Cre^+^ mice compared with aging WT mice (Figure 2E). These findings are consistent with the structural deterioration of endothelial cells in the aging kidneys of Atg7*^flox/flox^*;Tie2-Cre^+^ mice.

Taken together, these findings indicate that autophagy deficiency in glomerular EC in renal aging induced the detachment of EC with the disruption of GBM assembly and exacerbated podocyte loss and apoptosis, which contributed to microalbuminuria.

### 3.3. EC-Specific Atg7 Deletion Increased Ferritin Accumulation in Renal Aging

**Figure 3:**
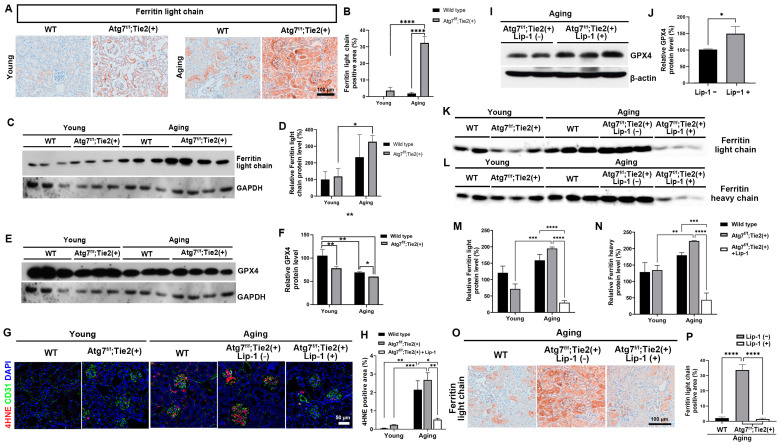
Liproxstatin-1 treatment increases the glutathione peroxidase (GPX4) and decreases the ferritin accumulation in the renal aging of Atg7*^flox/flox^*;Tie2-Cre^+^ mice. (**A**) Representative 3,3′-diaminobenzidine (DAB) staining for ferritin light chain. Scale bars, 100 μm. (**B**) Quantification of ferritin light chain-positive areas (%). (**C**) Representative immunoblots and densitometry for the expression of ferritin light chain. (**D**) Relative protein levels of ferritin light chain (%). (**E**,**F**) Representative immunoblots and densitometry for the expression and relative protein levels of GPX4 (%). (**G**) Representative immunofluorescent staining for 4HNE (red) and CD31 (green). Scale bars, 50 μm. (**H**) Quantification of 4HNE positive areas (%). (**I**,**J**) Representative immunoblots and densitometry for the expression and relative protein levels of GPX4 (%). (**K**–**N**) Representative immunoblots and densitometry for the expression of ferritin light and heavy chain sand relative protein levels of ferritin light and heavy chains (%). (**O**) Representative 3,3′-diaminobenzidine (DAB) staining for ferritin light chain. Scale bars, 100 μm. (**P**) Quantification of ferritin light chain-positive areas (%). Values are means ± SEM. *, *p* < 0.05; **, *p* < 0.01, ***; *p* < 0.001, ****; *p* < 0.0001.

Aging is associated with the imbalance of iron metabolism and increased stores of iron in tissues [37]. The accumulated iron induces deleterious effects on cellular functions and contributes to aging by ferroptosis [37]. We examined the effect of endothelial autophagy on iron accumulation in the kidneys during aging. Interestingly, the accumulation of ferritin, a major intracellular protein that stores iron, in kidney tissue was substantially increased in aging WT mice compared with young mice and was substantially increased in the aging kidneys of Atg7*^flox/flox^*;Tie2-Cre^+^ mice (Figure 3A,B). The accumulation of ferritin in autophagy-deficient aging kidneys was observed in ECs as well as the glomerulus and tubules (Figure 3A,B). The protein expression of ferritin was also increased in the aging kidneys of Atg7*^flox/flox^*;Tie2-Cre^+^ mice (Figure 3C,D). These findings provide evidence that EC-specific Atg7 deletion accelerates the accumulation of ferritin in the tissue of aging kidneys.

Ferroptosis is a form of regulated cell death associated with the iron-dependent accumulation of lipid hydroperoxides, which are a type of ROS. This occurs when glutathione-dependent lipid peroxide repair systems are compromised. In the context of the increased accumulation of ferritin in EC-specific Atg7-deficient aging kidney tissue based on our data (Figure 3A), we hypothesized that endothelial autophagy deficiency in aging kidneys leads to ferroptotic cell death in kidney tissue.

Glutathione peroxidase 4 (GPX4), a lipid repair enzyme, is the central regulator of ferroptosis [38]. Immunoblot assay showed the decreased immunoreactivity of GPX4 in the aging Atg7*^flox/flox^*;Tie2-Cre^+^ mice compared with aging WT mice (Figure 3E,F). Immunofluorescence staining revealed that the lipid peroxidation product, 4-HNE, was increased in the aging Atg7*^flox/flox^*;Tie2-Cre^+^ mice compared with aging WT mice (Figure 3G,H). These findings indicate the activation of the ferroptotic pathway in endothelial autophagy deficiency in renal senescence.

### 3.4. Liproxstatin-1 Attenuated Renal Senescence from Endothelial Autophagy Deficiency by Regulating the Ferroptosis Pathway In Vivo

Liproxstatin-1 has been reported to inhibit ferroptosis by inhibiting lipid autoxidation and attenuating GPX4 inactivation [38,39]. We examined whether treatment with liproxstatin-1, a ferroptosis inhibitor, can regulate the ferroptotic pathway and attenuate renal senescence in the aging Atg7*^flox/flox^*;Tie2-Cre^+^ mice.

Treatment with liproxstatin-1, a ferroptosis inhibitor, increased the protein expression of GPX4 (Figure 3I,J) and decreased the lipid peroxidation product, 4-HNE, in the aging kidneys of Atg7*^flox/flox^*;Tie2-Cre^+^ mice (Figure 3G,H). Consequently, liproxstatin-1 treatment also decreased the accumulation of L-ferritin and H-ferritin in the aging kidneys of Atg7*^flox/flox^*;Tie2-Cre^+^ mice (Figure 3K–P).

**Figure 4:**
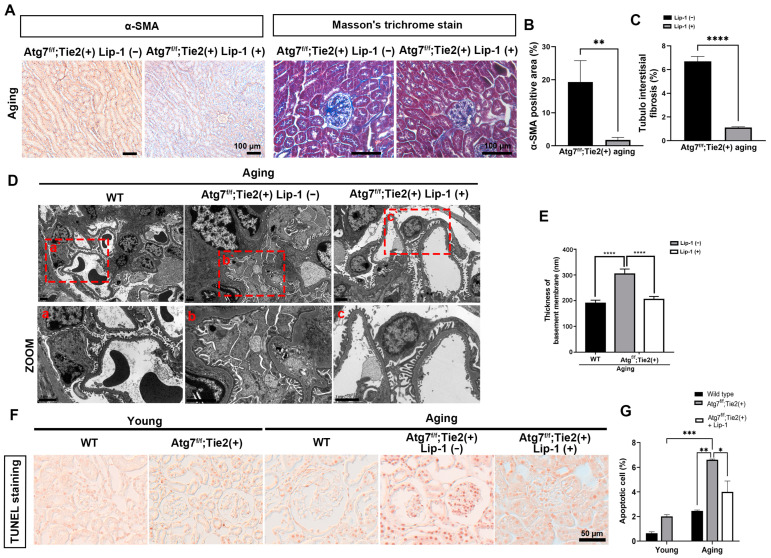
Attenuated renal aging in endothelial autophagy-deficient aging kidneys by liproxstatin-1. (**A**) Representative 3,3′-diaminobenzidine (DAB) staining for α-SMA and Masson’s trichrome staining. Scale bars, 100 μm. (**B**) Quantification of α-SMA positive areas (%). (**C**) Quantification of tubulointerstitial fibrosis (%). (**D**) Representative TEM images showing the apoptotic podocytes and structural integrity of slit diaphragms. The thickness of the glomerular basement membrane increased in aging Atg7*^flox/flox^*;Tie2-Cre*^+^* mouse kidneys (**b’**) compared to aging WT kidneys (**a’**). Liproxstatin-1 treatment attenuated the increased thickness of the glomerular basement membrane in aging Atg7*^flox/flox^*; Tie2-Cre^+^ mouse kidneys (**c’**). (**E**) Quantification of glomerular basement membrane thickness (nm). (**F**) Immunostaining for the expression and (**G**) quantification of TUNEL-positive cells. Scale bars, 50 μm. Values are means ± SEM. *, *p* < 0.05; **, *p* < 0.01; ***, *p* < 0.001; ****, *p* < 0.0001.

After treatment with liproxstatin-1, Masson’s trichrome staining and immunohistochemical staining revealed a substantial decrease in ECM deposition and decreased the expression of α-SMA in the glomerulus and tubulointerstitium (Figure 4A–C), indicating decreased renal fibrosis as a phenotype of renal senescence. Liproxstatin-1 treatment also attenuated the increased thickness of GBM in endothelial autophagy-deficient aging kidneys (Figure 4D,E). TUNEL staining revealed that the increased apoptotic cell death in the glomerulus and tubules of the aging kidneys of Atg7*^flox/flox^*;Tie2-Cre^+^ mice was decreased after liproxstatin-1 treatment (Figure 4F,G). Taken together, liproxstatin-1 treatment increased GPX4 and reduced lipid peroxidation. This regulation of the ferroptosis pathway resulted in decreased ferritin accumulation and consequently attenuated renal senescence in endothelial autophagy-deficient kidneys.

### 3.5. Disruption of the Endothelial Barrier by Autophagy Inhibition with Iron Loading and Oxidative Stress Accelerated the Ferroptotic Cell Death in the Human Kidney Organoid In Vitro Model

Based on in vivo experiments demonstrating that EC-specific Atg7 deletion induced the detachment of ECs with the disruption of the GBM assembly and activated the ferroptotic pathway in aging kidneys, we hypothesized that the increased iron loading and oxidative stress during renal senescence resulted in the disruption of the endothelial barrier in autophagy deficiency, which leads to the leakage of iron and oxidative stress from the capillary lumen in nephrons and accelerated ferroptotic cell death in kidneys.

**Figure 5:**
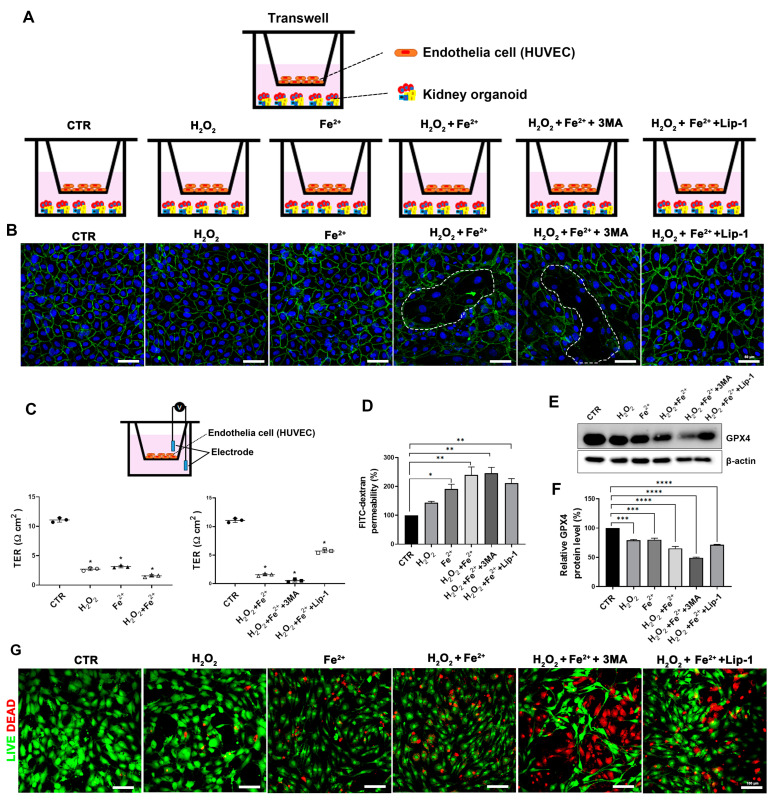
The liproxstatin-1 regulation of ferroptosis in endothelial cells in vitro. (**A**) Schematic diagram of ferroptosis model using kidney organoids in vitro. (**B**) Immunofluorescent tight junction staining in HUVECs. Distribution of the tight junction (VE-cadherin) and DAPI showing the distribution of HUVEC nuclei. The White dashes indicate damaged tight junction. Scale bars, 50 μm. (**C**) Transwell assay; TEER measurement of endothelial cells. (**D**) FITC-dextran permeated in the medium was evaluated to assay the permeability of HUVECs in each group. (**E**) Representative immunoblots and densitometry of the expression of GPX4. (**F**) Relative protein levels of GPX4 (%). (**G**) Representative images of live/dead staining. Scale bars: 100 μm. Values are means ± SEM. *, *p* < 0.05; **, *p* < 0.01; ***, *p* < 0.001; ****, *p* < 0.0001.

To examine this hypothesis, we made a Transwell co-culture system of ECs and kidney organoids (Figure 5A). Kidney organoids were cultured on the bottom of culture plates with ECs, and HUVECs were cultured on the Transwell inserts and placed into the culture plates (Figure 5A).

HUVECs cultured on the Transwell inserts were treated with H_2_O_2_ (5 mM), Fe^2+^ (500 μM), and H_2_O_2_ (5 mM) + Fe^2+^ (500 μM) (Figure 5A). The selected dose was determined according to cell viability (Figure S1A,B).

First, we analyzed the vascular continuity and endothelial function of HUVECs cultured on the Transwell inserts. Confocal microscopy analysis showed that the control HUVECs (not exposed to H_2_O_2_ and Fe^2+^) had clear, tight, and continuous VE-cadherin distributions in cell–cell junctions (Figure 5B and Figure S2). After exposure to H_2_O_2_ and Fe^2+^ for 24 h, VE-cadherin expression declined, and their continuity was lost, which was accelerated in the group treated with class III phosphatidylinositol 3-kinase inhibitor 3-methyladenine (3MA), an autophagy inhibitor (Figure 5B and Figure S2).

We measured the endothelial barrier function by assaying the permeability of HUVECs. The TER measurement is useful as a parameter for the determination of vascular permeability. The TER value of HUVEC monolayers was decreased after exposure to H_2_O_2_ + Fe^2+^ (Figure 5C) and decreased further after 3MA treatment (Figure 5C).

We also analyzed the relative permeability of HUVECs by measuring FITC dextran. HUVECs were cultured as tight monolayers and added to an upper Transwell chamber in FITC-labeled dextran (2 mg/mL; 70 kDa) for 2 h. The FITC-dextran level of the lower chamber was increased in the group treated with H_2_O_2_ + Fe^2+^ and increased further in the group treated with 3MA (Figure 5D).

Liproxstatin-1 treatment recovered the protein expression of GPX4 in HUVECs, which was decreased after exposure to H_2_O_2_ + Fe^2+^ and 3MA (Figure 5E,F). Liproxstatin-1 attenuated the disruption of junctional VE-cadherin continuity (Figure 5B and Figure S2) and recovered endothelial barrier function (Figure 5C,D).

We then examined live/dead staining. This showed that the number of dead cells increased after exposure to H_2_O_2_ + Fe^2+^ and further increased by 3MA (Figure 5G). Cell viability was recovered by liproxstatin-1 treatment consistent with the level before 3MA treatment (Figure 5G).

These findings indicate that iron loading and oxidative stress induce the disruption of the endothelial barrier and increase vascular permeability by activating the ferroptotic pathway, which is accelerated by the inhibition of autophagy.

**Figure 6:**
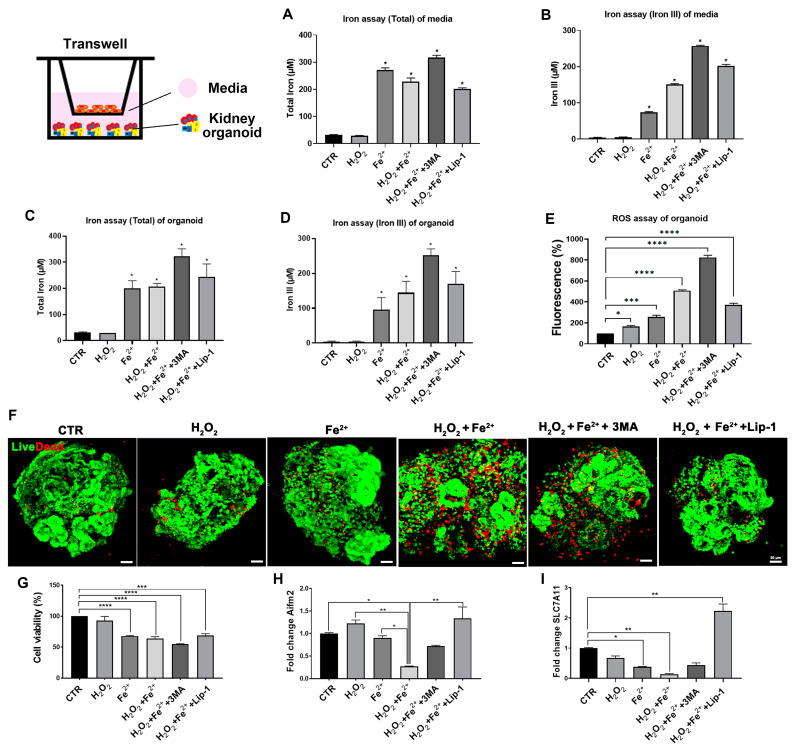
The liproxstatin-1 regulation of ferroptosis in kidney organoid–endothelial cells in vitro using the Transwell system. (**A**–**D**) The iron level (total and iron III) in media of the lower compartment and kidney organoids was detected using an iron assay kit. (**E**) Measurement of fluorescence intensity by DCFDA/H2DCFDA. (**F**) Representative images of live/dead staining. Scale bars: 50 μm. (**G**) Kidney organoid viability according to the CellTiter-Glo 3D Reagent Cell Viability Assay. (**H**,**I**) qRT-PCR analysis of Airm2 and SLC7A11 gene (*n* = 3). Values are means ± SEM. *, *p* < 0.05; **, *p* < 0.01; ***, *p* < 0.001; ****, *p* < 0.0001.

Next, we examined how the disruption of the endothelial barrier induced by iron loading and oxidative stress affected kidney organoids cultured on the bottom of culture plates in the Transwell co-culture system. We postulated that the disruption of the endothelial barrier and increased permeability may result in an increase in the level of iron in the culture medium as well as kidney organoids, leading to oxidative stress in kidney organoids. We examined the iron and ROS levels in the culture medium and kidney organoids in the Transwell co-culture system. The iron levels in the culture medium increased after H_2_O_2_ + Fe^2+^ treatment and were further increased by 3MA (Figure 6A,B). Liproxstatin-1 treatment recovered the levels of iron and ROS to the level before 3MA treatment (Figure 6A,B).

The levels of iron and ROS in the kidney organoids increased after H_2_O_2_ + Fe^2+^ treatment and were further increased by 3MA (Figure 6C–E). Liproxstatin-1 treatment recovered the levels of iron and ROS to the level before 3MA treatment (Figure 6C–E).

We then examined cell viability (Figure 6F,G). Live/dead staining showed that the number of dead cells increased after exposure to H_2_O_2_ + Fe^2+^ and further increased by 3MA (Figure 6F). Cell viability was recovered by liproxstatin-1 treatment consistent with the level before 3MA treatment (Figure 6F). Similar findings were observed in cell viability assessed by CCK-8 (Figure 6G). The mRNA expression of apoptosis-inducing factor mitochondria associated 2 (AIFM2) and solute carrier family 7 member 11 (SLC7A11), the key regulators in ferroptosis (antiferroptotic pathway) was decreased after exposure to H_2_O_2_ + Fe^2+^ and decreased further by 3MA treatment. Liproxstatin-1 treatment recovered the gene expression of AIFM2 and SLC7A11 (Figure 6H,I).

Taken together, our findings show that the inhibition of autophagy, iron loading, and oxidative stress in ECs disrupted the endothelial barrier and increased vascular permeability. The leakage of iron and oxidative stress from the Transwell inserts accelerated ferroptotic cell death in kidney organoids cultured on the bottom of culture plates. This provides evidence that endothelial deficiency leads to ferroptosis in nephrons in renal senescence.

### 3.6. EC-Specific Atg7 Deletion Increased Oxidative Stress and Upregulated the NLRP3 Inflammasome Signaling Pathway in Renal Senescence

Excess iron can lead to the production of ROS produced by the Fenton reaction, which results in oxidative stress in the aging process [19,37,40]. ROS are potential signals for NLRP3 inflammasome activation. NLRP3 and other members of the NLRs are involved in innate immune responses [41]. NLRP3 forms a protein complex, the inflammasome, which induces caspase-1 activation that results in the maturation and secretion of proinflammatory cytokines such as IL-1β and IL-18 [42]. Thus, the NLRP3 inflammasome signaling pathway regulates a variety of host innate immune defense pathways in response to pathogens or damage-associated molecular patterns by microbial and nonmicrobial stimuli [42].

Based on our in vitro results, we hypothesized that the NLRP3 inflammasome signaling pathway is activated by excess iron leakage from capillaries after the disruption of the endothelial barrier in autophagy-deficient aging kidneys and increased ROS production during renal senescence.

**Figure 7:**
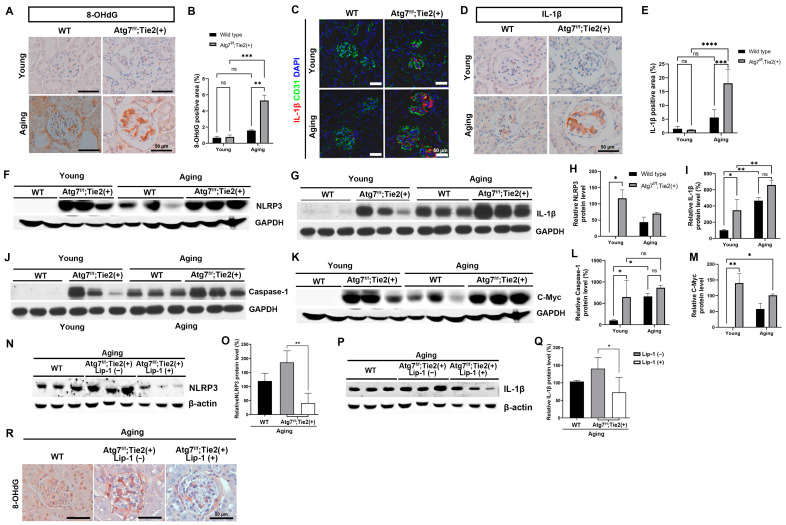
Increase in oxidative stress and the activation of the inflammasome pathway in renal aging of Atg7*^flox/flox^*;Tie2-Cre*^+^* mice. (**A**) Representative 3,3′-diaminobenzidine (DAB) staining for 8-OHdG. Scale bars, 50 μm. (**B**) Quantification of 8-OHdG positive areas (%). (**C**) Representative immunofluorescent staining for IL-1β (red) and CD31 (green). Scale bars, 50 μm. (**D**) Representative DAB staining for IL-1β. Scale bars, 50 μm. (**E**) Quantification of IL-1β positive areas (%). (**F**) Representative immunoblots and densitometry for the expression of NLRP3. (**G**) Representative immunoblots and densitometry for the expression of IL-1β. (**H**,**I**) Relative protein levels of NPRP3 and IL-1β (%). (**J**) Representative immunoblots and densitometry for the expression of caspase-1. (**K**) Representative immunoblots and densitometry for the expression of c-Myc. (**L**,**M**) Relative protein levels of Caspase-1 and c-Myc (%). (**N**,**O**) Representative immunoblots and densitometry for the expression and relative protein levels of NPRP3. (**P**,**Q**) Representative immunoblots and densitometry for the expression and relative protein levels IL-1β. (**R**) Representative 3,3′-diaminobenzidine (DAB) staining for 8-OHdG. Scale bars, 50 μm. Values are means ± SEM. *, *p* < 0.05; **, *p* < 0.01, ***; *p* < 0.001, ****; *p* < 0.0001, ns: not significant.

To examine this hypothesis, we performed immunohistochemical analysis, which showed that the increased immunoreactivity of 8-hydroxy-2′-deoxyguanosine (8-OHdG), a marker of oxidative DNA damage, was observed in the glomerulus of aging Atg7*^flox/flox^*;Tie2-Cre^+^ mice compared with aging WT mice (Figure 7A,B). Immunofluorescence and immunohistochemical staining revealed the activation of IL-1β, a protein that is downstream of the NLRP3 inflammasome, in the glomerulus of the aging Atg7*^flox/flox^*;Tie2-Cre^+^ mice (Figure 7C–E). Immunoblot assays from kidney tissue revealed that Atg7 deficiency in ECs induced the NLRP3/caspase-1/IL-1β signaling pathway in aging kidneys (Figure 7F–M).

A previous study demonstrated that the accumulation of Myc and upregulated Myc target gene resulting from IL-1β stimulation was necessary for renal progressive tubulointerstitial fibrosis, a typical histologic feature of renal aging [43]. In this study, the protein expression of c-Myc was substantially increased in aging Atg7*^flox/flox^*;Tie2-Cre*^+^* mice. These findings suggested that the upregulated NLRP3/Caspase-1/IL-1β signaling pathway in endothelial autophagy-deficient aging kidneys resulted in the accumulation of c-Myc, which may enhance renal tubulointerstitial fibrosis (Figure 7F–M).

Our in vitro experiment showed that liproxstatin-1 treatment attenuated the disruption of the endothelial barrier and recovered the level of iron to that before 3MA treatment (Figure 5B–D). We examined whether liproxstatin-1 treatment attenuated the NLRP3 inflammasome signaling pathway during renal senescence in vivo. Immunoblot assays showed that the expression of NLRP3 and IL-1β decreased after liproxstatin-1 treatment in the aging kidneys of Atg7*^flox/flox^*;Tie2-Cre^+^ mice (Figure 7N–Q). Increased oxidative DNA damage (8-OHdG) was also attenuated by liproxstatin-1 treatment in the aging kidneys of Atg7*^flox/flox^*;Tie2-Cre^+^ mice (Figure 7R).

Taken together, these findings suggest that a deficiency in endothelial autophagy increases oxidative stress in nephrons and activates the NLRP3 inflammasome signaling pathway during renal senescence. Furthermore, the restoration of the endothelial barrier by liproxstatin-1 attenuates the NLRP3 inflammasome signaling pathway.

## 4. Discussion

In this study, we demonstrated how endothelial autophagy interacts with ferroptosis and the NLRP3 inflammasome signaling pathway and regulates renal aging. Our data showed that the deficiency of endothelial autophagy can result in an imbalance of iron metabolism. Increased oxidative stress with aging disrupts the endothelial barrier and increases vascular permeability. The leakage of iron from capillaries with oxidative stress during aging accelerates the ferroptotic cell death of kidney tissue and activates the NLRP3 inflammasome signaling pathway.

Previous studies have investigated the role and mechanisms of autophagy in renal aging. The podocyte-specific deletion of Atg5 resulted in mitochondrial damage, endoplasmic reticulum stress, and the accumulation of oxidized protein aggregates in podocytes, which promoted aging-related cellular dysfunction and structural changes such as proteinuria, the loss of podocytes, and glomerulosclerosis [8]. Autophagy impairment in proximal tubular cells in aging mice results in kidney dysfunction and tubulointerstitial fibrosis, which are concomitant with mitochondrial dysfunction as well as damage to mitochondrial and nuclear DNA [9].

Our study provides evidence for another important mechanism by which autophagy affects renal aging, which is different from previous studies. We focused on ECs for the role of autophagy in renal aging, taking into account that vascular oxidative stress and chronic inflammation are common phenomena during senescence [44].

In this study, we chose an Atg7 mutant mouse model as an experimental model of aging kidneys among autophagy-related genes. Numerous autophagy-related genes are associated with the aging process [43]. Deficiency in Atg1, Atg7, Atg18, and Beclin 1 decreases the life span of the nematode Caenorhabditis elegans [43], and the deficient expression in Atg1, Atg8, and Sestrin1 reduces the life span of the fruit fly Drosophila melanogaster [45,46]. Among autophagy-related (ATG) genes, ATG7 encodes an E1-like enzyme, which is a critical factor that initiates classic autophagy reactions [47]. ATG7 activates ATG12 before its conjugation to ATG5 and promotes the formation and extension of autophagosome membranes [47]. ATG7 also contributes to the lipidation of the protein LC3-I with phosphatidylethanolamine to generate LC3-II and drives the cardinal stages of classical degradative autophagy [48]. ATG7 also plays a critical role in innate immunity via LC3-associated phagocytosis, unconventional protein secretion, receptor recycling, the exocytosis of secretory granules, and the modulation of p53-dependent cell cycle arrest and apoptosis, which is associated with the aging process [47,48,49]. Recent studies have identified the relationship of ATG7 with aging-related diseases [47,50,51]. Furthermore, defective ATG7-mediated autophagy causes human disease [50]. Therefore, we chose an Atg7-deficient animal model as an aging model.

**Figure 8:**
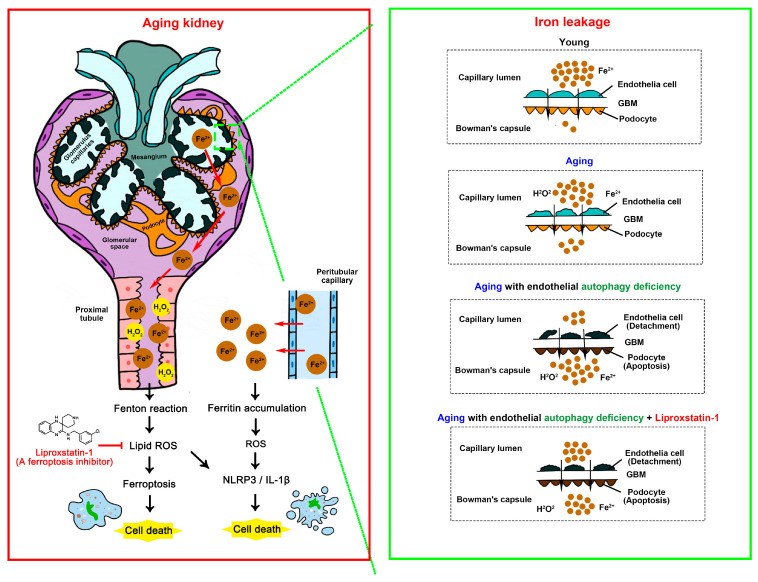
Schematic overview.

One of the major findings of our study is that endothelial autophagy deficiency in the imbalance of iron metabolism and increased oxidative stress during renal aging disrupted the endothelial barrier and increased vascular permeability, which induced vascular leakage from capillaries. In aging, life-long exposure to environmental factors and countless interactions with infectious agents leads to chronic inflammation, so-called inflammaging, and proinflammatory mediators are increased in serum [52]. The vascular leakage of iron as well as proinflammatory cytokines from capillaries such as glomerular capillaries and peritubular capillaries into the urinary space and renal tubules may increase the accumulation of ROS and ferritin in glomerulus and tubules, which induce oxidative stress and ferroptotic cell death as well as the NLRP3 inflammasome signaling pathway (Figure 8).

A previous study reported the impact of vascular damage and plasma leakage on glomerular injury in Alport syndrome, a genetic kidney disease [53]. Using Col4A3-deficient mice, they showed the disruptions and progressive disintegration of the GBM-induced vascular leakage from glomerular capillaries into Bowman’s space, which triggers cellular and fibrocellular crescent formation and develops a progressive glomerulopathy [53].

Given the importance of maintaining EC homeostasis and the function of the endothelial barrier in renal aging, our data highlight the role of endothelial autophagy in regulating these processes.

Another major finding of our study is that endothelial autophagy interacts with ferroptosis in renal aging. Our data suggest that chronic inflammation by the vascular leakage of proinflammatory mediators from capillaries by endothelial autophagy deficiency during renal aging induces iron dyshomeostasis and increases iron storage in the glomerulus and renal tubules, which triggers ferroptosis by the production of ROS through the Fenton reaction (Figure 8).

The basic process of aging is proposed to be a consequence of cumulative oxidative damage caused by ROS [37]. Aging is associated with the imbalance of iron metabolism and the accumulation of iron stores in tissues, which is a conserved phenomenon [37]. Excess iron can result in ferroptosis through the production of ROS through the Fenton reaction. Ferroptosis is an iron-dependent regulated cell death characterized by the production of ROS from accumulated iron and lipid peroxidation [17,18]. Ferroptosis is mainly caused by deficits in the production of reduced glutathione or by the dysfunction of GPX4, which are ROS eliminators [19]. Our in vitro data demonstrated that autophagy deficiency accelerated EC death in iron-loading conditions and increased oxidative stress, which was rescued by liproxstatin-1 treatment. Our data show the link between autophagy and ferroptosis in ECs during renal aging.

Another major finding in our study was that endothelial autophagy interacts with the NLRP3 inflammasome signaling pathway in renal aging. In innate immunity, autophagy has been reported to play a pivotal role in regulating inflammasome activation and IL-1 family cytokine secretion by the removal of inflammasome-activating endogenous signals or the sequestration and degradation of inflammasome components [41]. This study suggests a link between autophagy in ECs and the NLRP3 inflammasome signaling pathway in renal aging from a different perspective. Our data indicated that increased oxidative stress in kidney tissue by vascular leakage of proinflammatory mediators from capillaries may trigger the NLRP3 inflammasome signaling pathway in glomerulus and tubules. Increased oxidative stress activates the NLRP3 inflammasome/caspase-1/IL-1β signaling pathway and induces the apoptosis of renal cells. Activated IL-1β stimulates the accumulation of c-Myc and upregulates the c-Myc target gene, which contributes to renal fibrosis in renal aging. Our data link the mechanism of autophagy in ECs with renal fibrosis and apoptotic cell death during renal aging.

Our data also showed that liproxstatin-1 treatment attenuated the progression of renal aging in endothelial autophagy deficiency. Based on these experiments, we propose three mechanisms through which liproxstatin-1 contributes to the anti-aging of kidneys. First, liproxstatin-1 treatment recovered the structure and function of the disrupted endothelial barrier by increasing GPX4 and reducing ferroptotic EC death, which decreased vascular leakage. Second, the systemic administration of liproxstatin-1 diffuses into the glomerulus and renal tubules. An increase in GPX4 reduces lipid peroxidation, which rescues renal cells from ferroptosis. Third, the recovered endothelial barrier by liproxstatin-1 treatment decreases the leakage of proinflammatory cytokines from the capillaries into the urinary space and kidney tissue, which results in the downregulation of the NLRP3 inflammasome signaling pathway. This may contribute to cell death and fibrosis in kidney tissue during renal aging.

In conclusion, our data showed that endothelial autophagy plays a protective role in renal aging through the regulation of ferroptosis and the NLRP3 inflammasome pathway. Our data also demonstrate that ferroptosis regulation by liproxstatin-1 attenuates the progression of renal aging from endothelial autophagy deficiency, and this may be a potential therapeutic target to reduce cellular injury from renal aging (Figure 8).

## Data Availability

The data that supports the findings of this study are available in the Supplementary Materials of this article.

## Supplementary Materials

The following supporting information can be downloaded at: https://www.mdpi.com/article/10.3390/antiox13080886/s1, Figure S1: The selected dose was determined according to cell viability; Figure S2: Immunofluorescent tight junction staining in HUVECs.
