# Supplementary material for: The Impairment of Endothelial Autophagy Accelerates Renal Senescence by Ferroptosis and NLRP3 Inflammasome Signaling Pathways with the Disruption of Endothelial Barrier
Source: Antioxidants (Basel). 2024 Jul 23;13(8):886. doi: 10.3390/antiox13080886 (PMC11351978; doi:10.3390/antiox13080886)
Supplement: Supplementary file 1 [file antioxidants-13-00886-s001.zip › antioxidants-3087531-supplementary.pdf]

Supplementary Materials:

Supplementary Figure S1. The selected dose was determined according to cell viability.

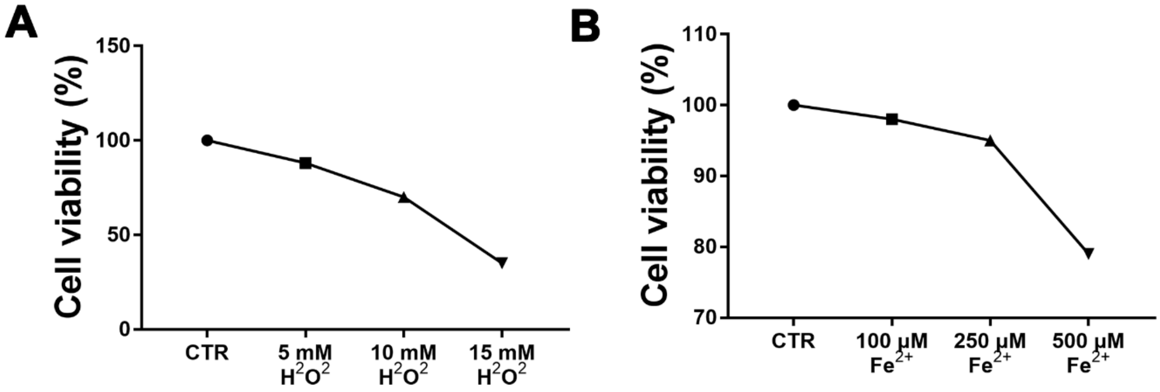

(**A,B**) Cell viability analysis was performed with 5 to 15 mM H^2O^2 and 100 to 500 µM Fe^{2+} to select the dose.

Supplementary Figure S2. Immunofluorescent tight junction staining in HUVECs.

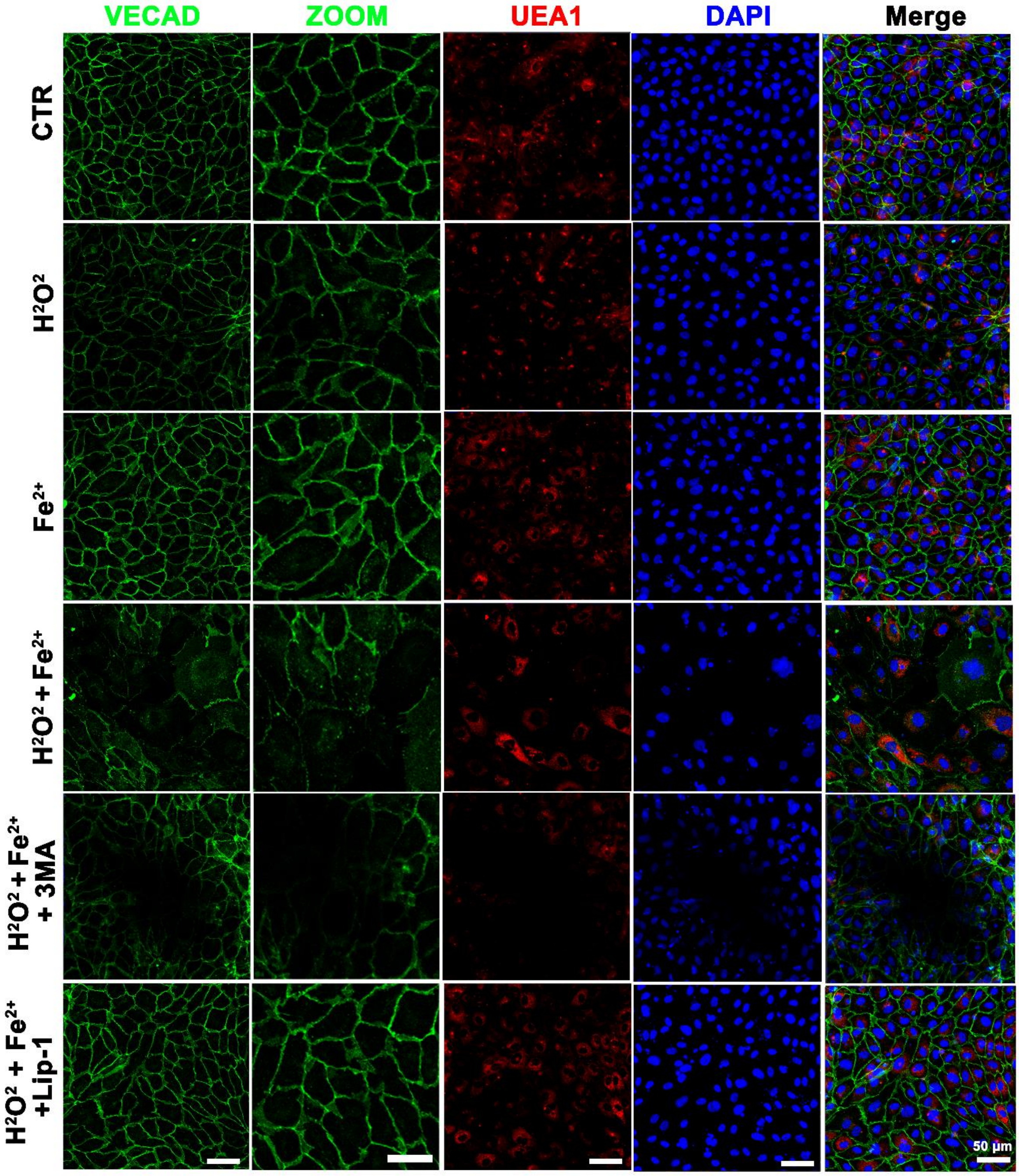

Distribution of the tight junction (VE-cadherin), Ulex europaeus agglutinin-1 (UEA-1) and DAPI showing the distribution of HUVEC nuclei. Scale bars, 50 μm.
